# The School Doctor's Outfit

**Published:** 1915-07-31

**Authors:** 


### THE SCHOOL DOCTOR'S OUTFIT.

### I.—The Lesson of Experience.

WHEN the medical inspector of schools starts his work he usually limits himself, so far as outfit is concerned, to a few instruments carried in a hand-bag. Later on, when his experience has increased and he becomes interested in various "specialities" and attempts special investigations, he finds the need for a larger and more comprehensive armamentarium. A few words on the choice and the selection of instruments may, therefore, not be out of place here. We will start by presuming that the school doctor is interested in his work and that he has taken it up as a career. Not all men or women do that. Indeed, it is a regrettable certainty that most men and women look upon school medical inspection as a blind alley, or a step on the ladder towards a medical officer of health's post. This is regrettable, because school medical inspection has come to stay. It is not to be looked upon as a temporary time-serving job; to be successful at it the medical inspector must be enthusiastic, and he or she must clearly understand that, although it may legitimately be classed as belonging to the category of public health work, it is different from the ordinary routine work of the medical officer of health.

### The Diploma of Public Health.

There has in the past been, and there exists in some quarters at present, an unfortunate tendency to look upon the public health part of the school doctor's work as the chief and most essential part of his services. The attitude of the Board of Education is perhaps responsible for this tendency. Yet it is clear that the man or woman who is specially well qualified to undertake public health work may make a very indifferent school doctor if he or she does not possess equally high qualifications in pediatrics. It must not be forgotten that the officers of the Board of Education are not primarily specialists in the diseases and psychology of children; they are, indeed, without that experience which it is logical to demand of school doctors, and it is only human that they should, therefore, exalt their own speciality—public health work—at the expense of the more important work that the school doctor has to undertake. The insistence by some educational authorities on the possession of a D.P.H. by applicants for school medical inspectorships is to be deplored, simply because the possession of that diploma—an excessively overrated qualification, by the way—is no guarantee that the applicant is a suitable candidate for the work he or she will be called upon to do. It is far more to be desired that applicants for school medical inspectorships shall have attended for a year at least at a children's hospital, that they shall have taken one of the specialist's qualifications in medicine or surgery which presuppose at least an acquaintance with the special diseases of children, and that they shall have some knowledge—special knowledge—of ophthalmology, otology, orthopædics, or bacteriology. The D.P.H. does not ensure this; at the most it is evidence that the candidate has attended a course of lectures, that he has worked in a laboratory, written a few reports, and is able to test the purity of water and foodstuffs—which tests he will never be called upon to apply in ordinary school work. It is, therefore, far preferable to let the medical officer of health do the purely sanitary part of school work and to allow the school doctor to apply himself to the investigation of special matters that demand knowledge and aptitude such as the average medical officer of health, who is ordinarily divorced from all clinical experience, does not possess.

This excursion does not concern the school doctor's outfit, but may be excused by way of preface, as it has some bearing upon the question of necessary apparatus and instrumentarium. If the school doctor goes to his work obsessed by the idea that he is primarily a sanitary inspector, he will have to carry with him instruments for testing the purity of the air, the direction and rate of air currents, the pressure in the water pipes, and materials for testing the purity of the drinking water supplied at the points. No school doctor does that. Even when he has to give a report on the sanitary state of the school buildings he contents himself with observation and such simple tests as every qualified medical man should know how to apply.

We will start, therefore, by supposing that the school doctor goes to school with the intention of medically examining children, and as a side issue to investigate certain points in which he takes a professional and practical interest. For his work he needs certain instruments, and to carry these to the school he requires a bag. The school doctor's bag is, therefore, the first consideration.

### The Doctor's Bag.

I do not know of any instrument-maker who has yet manufactured a suitable school doctor's, —or, indeed, any doctor's—bag. After wasting money on several patent and semi-patent contrivances, advertised to possess all the requirements of an ideal doctor's bag, I went to a trunk-makers and provided myself with a wide-mouthed, wide-angled kit-bag, of good leather, with strong lock and side clasps. This bag measures 18 inches by 6 by 13, and, modified by the maker in accordance with instructions, has served me usefully for several years, at a cost some 20 per cent. below what the surgical instrument-makers would have charged for a similar article. I had it lined inside with non-absorbent, easily-cleaned thin rubber cloth, fitted with side pockets, and with an upper tray on a light steel foundation, and had the corners strengthened by leather overlays.

In this bag I carry the following instruments and apparatus. This selection I have found suitable for all routine work; personal predilection will suggest modifications to individual inspectors according to their interest and taste.

*Tape-measure.*—This is a spring tape-measure, graduated on the metric system, made of strong tape, and enclosed in a steel spring casing. Wire steel measures are unsuitable, since they are apt to cut the flesh if not carefully adjusted, while they cannot be so accurately applied. No measurements should ever be taken in inches. It is still common for school doctors to give their measurements in the unscientific and absurd inches and feet; for purposes of comparison these measurements are almost useless, since to test them one has laboriously to convert them into the metric system. Weighing is now usually done on machines that are graduated according to the metric system, and it is absurd to employ a different scale for chest and arm measurements.

*Callipers.*—I use a Martin's pelvimeter, graduated in fractions of a centimetre, and find this the most useful instrument for taking head and chest measurements in anthropometric investigations. Special callipers for children are listed in all catalogues, but they are usually too small for the elder children, and never very strong. A Martin's pelvimeter serves for all such purposes, and is strongly made, easily adjustable, and unlikely to get out of order. I find the flexible cyrtometer, of soft metal, hinged, and graduated in centimetres, better than the leaden cyrtometer, which is heavy and easily breaks after being used for some time. Medium children's size is the most useful.

*Stethoscopes.*—I carry several, both binaural and wooden, the latter made to unscrew and lie flat in the upper tray. Wooden stethoscopes of the old-fashioned pattern are exceedingly useful for listening to heart murmurs, especially aortic and basal murmurs, but are less useful for listening to the lung sounds.

Dressing case of leather, fitted with trocar and cannula, Syme and Paget's knives, clinical thermometer (Kew tested half-minute), two pairs of Kocher's forceps, sinus forceps, fine forceps for extracting splinters, curved scissors, hypodermic syringe, with compressed drugs (eucaine, morphia, strychnine, and cocaine), surgical needles, some spools of horsehair and catgut (sterilised and ready for use when required), silver probes, and a few small capsules of amyl nitrite. You may never have to use any of these, but it is well to be prepared for all emergencies, and on several occasions the dressing-case has amply justified its inclusion in my bag. L.R.C.P.

(*To be continued.*)

